# Patient-Centered Economic Burden of Exudative Age-Related Macular Degeneration: Retrospective Cohort Study

**DOI:** 10.2196/49852

**Published:** 2023-12-08

**Authors:** Kyungseon Choi, Sang Jun Park, Sola Han, Yongseok Mun, Da Yun Lee, Dong-Jin Chang, Seok Kim, Sooyoung Yoo, Se Joon Woo, Kyu Hyung Park, Hae Sun Suh

**Affiliations:** 1 Department of Regulatory Science Graduate School Kyung Hee University Seoul Republic of Korea; 2 Institute of Regulatory Innovation Through Science Kyung Hee University Seoul Republic of Korea; 3 Department of Ophthalmology Seoul National University College of Medicine Seoul National University Bundang Hospital Seongnam Republic of Korea; 4 Health Outcomes Division College of Pharmacy University of Texas at Austin Austin, TX United States; 5 Department of Ophthalmology Kangnam Sacred Heart Hospital Hallym University College of Medicine Seoul Republic of Korea; 6 Department of Ophthalmology and Visual Science Yeouido St Mary’s Hospital, College of Medicine The Catholic University of Korea Seoul Republic of Korea; 7 Healthcare ICT Research Center Office of eHealth Research and Businesses Seoul National University Bundang Hospital Seongnam Republic of Korea; 8 Department of Ophthalmology Seoul National University College of Medicine Seoul National University Hospital Seoul Republic of Korea; 9 College of Pharmacy Kyung Hee University Seoul Republic of Korea

**Keywords:** blindness, age-related macular degeneration, economic burden, cost of illness, retrospective cohort study, common data model

## Abstract

**Background:**

Exudative age-related macular degeneration (AMD), one of the leading causes of blindness, requires expensive drugs such as anti–vascular endothelial growth factor (VEGF) agents. The long-term regular use of effective but expensive drugs causes an economic burden for patients with exudative AMD. However, there are no studies on the long-term patient-centered economic burden of exudative AMD after reimbursement of anti-VEGFs.

**Objective:**

This study aimed to evaluate the patient-centered economic burden of exudative AMD for 2 years, including nonreimbursement and out-of-pocket costs, compared with nonexudative AMD using the Observational Medical Outcomes Partnership (OMOP) Common Data Model (CDM).

**Methods:**

This retrospective cohort study was conducted using the OMOP CDM, which included 2,006,478 patients who visited Seoul National University Bundang Hospital from June 2003 to July 2019. We defined the exudative AMD group as patients aged >50 years with a diagnosis of exudative AMD and a prescription for anti-VEGFs or verteporfin. The control group was defined as patients aged >50 years without a diagnosis of exudative AMD or a prescription for anti-VEGFs or verteporfin. To adjust for selection bias, controls were matched by propensity scores using regularized logistic regression with a Laplace prior. We measured any medical cost occurring in the hospital as the economic burden of exudative AMD during a 2-year follow-up period using 4 categories: total medical cost, reimbursement cost, nonreimbursement cost, and out-of-pocket cost. To estimate the average cost by adjusting the confounding variable and overcoming the positive skewness of costs, we used an exponential conditional model with a generalized linear model.

**Results:**

We identified 931 patients with exudative AMD and matched 783 (84.1%) with 2918 patients with nonexudative AMD. In the exponential conditional model, the total medical, reimbursement, nonreimbursement, and out-of-pocket incremental costs were estimated at US $3426, US $3130, US $366, and US $561, respectively, in the first year and US $1829, US $1461, US $373, and US $507, respectively, in the second year. All incremental costs in the exudative AMD group were 1.89 to 4.25 and 3.50 to 5.09 times higher in the first and second year, respectively, than those in the control group (*P*<.001 in all cases).

**Conclusions:**

Exudative AMD had a significantly greater economic impact (*P*<.001) for 2 years on reimbursement, nonreimbursement, and out-of-pocket costs than nonexudative AMD after adjusting for baseline demographic and clinical characteristics using the OMOP CDM. Although economic policies could relieve the economic burden of patients with exudative AMD over time, the out-of-pocket cost of exudative AMD was still higher than that of nonexudative AMD for 2 years. Our findings support the need for expanding reimbursement strategies for patients with exudative AMD given the significant economic burden faced by patients with incurable and fatal diseases both in South Korea and worldwide.

## Introduction

### Background

Age-related macular degeneration (AMD) is a progressive retinal disorder that can lead to irreversible loss of central vision. AMD can be classified as dry or exudative (wet or neovascular). Exudative AMD is less common; however, it causes faster and more catastrophic vision loss than dry AMD. Anti–vascular endothelial growth factors (VEGFs) have demonstrated remarkable efficacy in averting blindness, converting once unavoidably blinding conditions into chronic diseases [1]. Anti-VEGFs are currently suggested as the primary treatment for exudative AMD [2], and 5 anti-VEGF agents (ie, ranibizumab, aflibercept, brolucizumab, bevacizumab, and faricimab) are clinically accessible. Photodynamic therapy with verteporfin, which was the first-line treatment in the past, is now used as adjuvant therapy for some subtypes of exudative AMD because of the lack of expectation of improvement in visual acuity [3]. All approved anti-VEGFs and modalities are quite expensive, at least US $500, and should be used repeatedly; however, bevacizumab, the only one used off-label, has demonstrated equivalent efficacy to ranibizumab [4] and is significantly inexpensive and widely used in patients [5].

In early randomized clinical trials, patients needed monthly injections in the first year of treatment under a fixed regimen. However, in real-world clinical practice, because of expensive out-of-pocket costs and strict reimbursement criteria, the fixed regimen was not affordable; thus, the treatment-and-extend regimen was introduced, showing similar efficient treatment outcomes with fewer injections [6]. Despite the introduction of more affordable treatment regimens [7], patients with exudative AMD still need 7 to 8 injections in the first year of treatment, which causes a substantial economic burden.

Although there are several studies [8-15] that have examined the economic burden of exudative AMD, they have some limitations that need to be addressed. First, most previous studies were conducted before the introduction of effective drugs such as anti-VEGFs, which have significantly affected the treatment landscape for exudative AMD. Second, previous studies were conducted through interviews, which may not provide an accurate evaluation of the true economic burden. Third, although a few studies analyzed real-world data, they only included reimbursed interventions and had a limited follow-up period of 1 year. In addition, exudative AMD is characterized by the common use of bevacizumab, an off-label drug, and nonreimbursed measurements, which add to the patients’ burden of disease. Given the intractable nature of exudative AMD, which requires long-term treatment and management, there is a critical unmet need to assess the long-term economic burden of patients with exudative AMD using real-world data, including nonreimbursed interventions.

### Objectives

Therefore, to fill this gap and generate real-world evidence to understand clinical, economic, and policy implications, we aimed to estimate the economic burden on patients with exudative AMD, including nonreimbursement costs up to the second year, using the real-world data from the Observational Medical Outcomes Partnership (OMOP) Common Data Model (CDM). Using electronic medical records (EMRs) transformed into the OMOP CDM of a major university hospital with a large number of patients with AMD is the best way to define real-world patients with exudative AMD and encompass drugs that are not reimbursed by the national health insurance in South Korea. This approach will facilitate the estimation of the economic burden, including nonreimbursed and out-of-pocket costs, from the patients’ perspective with a high quality of data.

## Methods

### Study Design and Data Source

This retrospective cohort study used the Observational Health Data Sciences and Informatics (OHDSI) OMOP CDM from Seoul National University Bundang Hospital (SNUBH), which is one of the largest and leading hospitals in South Korea. The OMOP CDM is a structured data model combined with standardized vocabulary. All medical and cost data were extracted, transformed, and loaded into the OMOP CDM version 5.3 following the OHDSI extract, transform, and load process and contained not only the diagnosis, drug exposure, and procedure occurrence for reimbursement but also all records for medical practice with costs by order and receipt regardless of reimbursement [16]. All data were verified using Automated Characterization of Health Information at Large-Scale Longitudinal Evidence Systems and double-checked by data analysts and clinicians [17,18].

### Study Population

**Figure 1:**
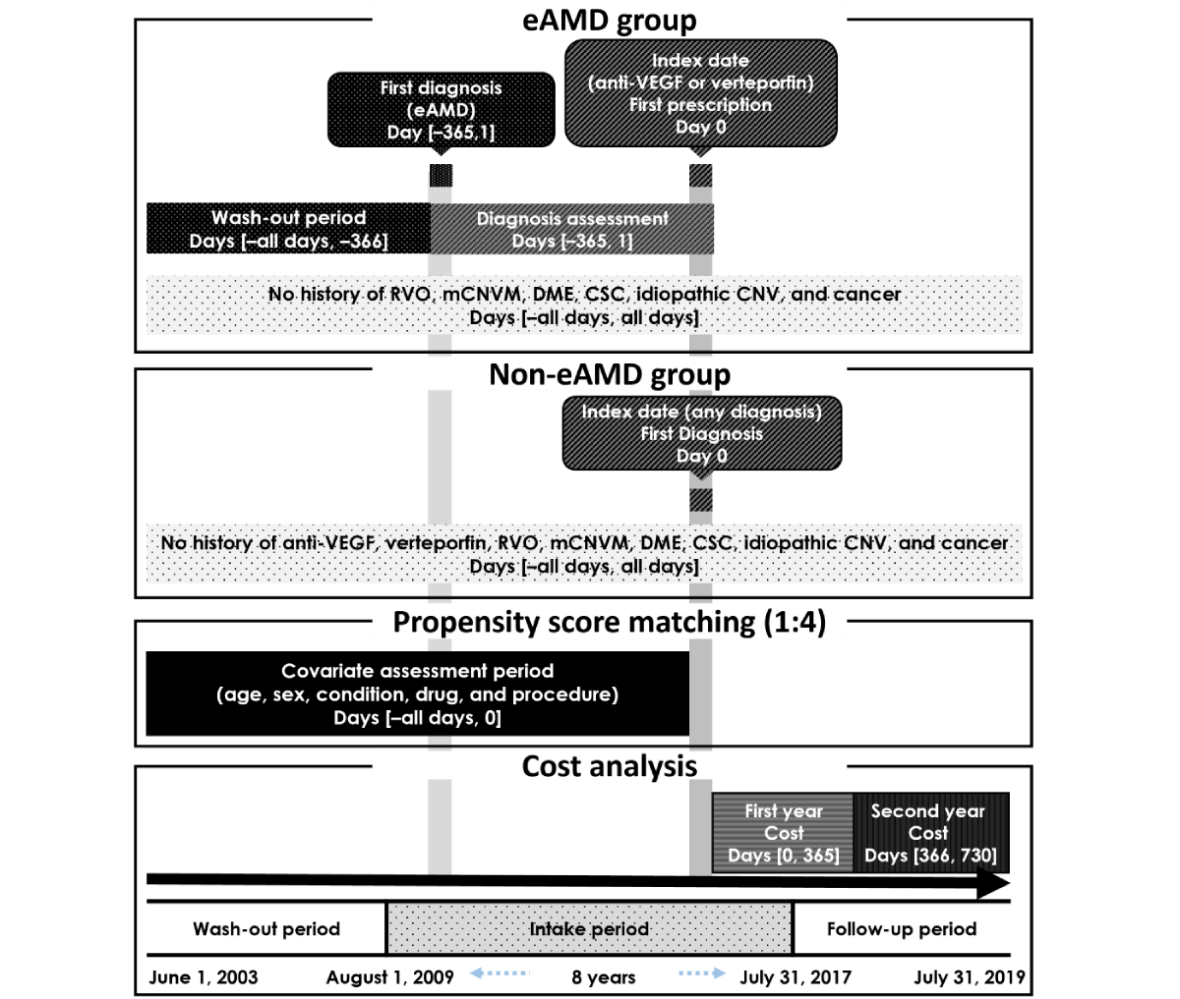
Analysis scheme. Data were included from June 2003 to July 2019. The intake period was defined as August 2009 to July 2017. The follow-up period was 2 years from the index date. CNV: choroidal neovascularization; CSC: central serous chorioretinopathy; DME: diabetic macular edema; eAMD: exudative age-related macular degeneration; mCNVM: myopic choroidal neovascular membrane; RVO: retinal vein occlusion; VEGF: vascular endothelial growth factor.

We analyzed the OMOP CDM database, which included 2,006,478 patients who visited SNUBH from June 2003 to July 2019. To ensure a 1-year wash-out period and a 2-year follow-up period, the intake period started in August 2009 and ended in July 2017. We established the eligibility criteria for the exudative and nonexudative AMD groups based on the opinions of ophthalmologists. For the exudative AMD group, the index date was the first prescription date for anti-VEGFs or verteporfin. The exudative AMD group was defined as follows: (1) a patient who was prescribed anti-VEGFs or verteporfin by an ophthalmologist for the first time between August 1, 2009, the day ranibizumab was approved for reimbursement by the National Health Insurance Service in South Korea, and July 31, 2017; and (2) a patient aged >50 years who had been diagnosed with exudative AMD within 1 year before the index date plus 1 day. Among these patients, we excluded those with cancer or other ophthalmic diseases using anti-VEGFs (choroidal neovascularization membrane, central serous chorioretinopathy, diabetic macular edema, and retinal vein occlusion). The nonexudative AMD group was defined as patients aged >50 years who had been diagnosed with any disease except exudative AMD between August 1, 2009, and July 31, 2017. We defined the index date for the nonexudative AMD group as the date of the first diagnosis of any disease to compare the outcomes of the 2 groups over time and investigate potential differences in disease progression and treatment efficacy. We also excluded patients with any of the following conditions: (1) those who were prescribed anti-VEGFs, (2) those diagnosed with cancer, and (3) those diagnosed with other ophthalmic diseases using anti-VEGFs (Figure 1).

### Outcomes

The economic burden of exudative AMD was measured by estimating the medical costs caused by any reason in the hospital in the first and second years after the index date (Figure 1). Medical costs were grouped into 4 categories (Textbox 1): total medical costs, reimbursement costs, nonreimbursement costs, and out-of-pocket costs. We estimated the incremental costs of the exudative AMD group in comparison with those of the nonexudative AMD group. The total medical cost was calculated as the sum of the reimbursement and nonreimbursement costs. The reimbursement cost includes all expenses only limited to reimbursed items, which comprise the costs of health care benefits from the National Health Insurance Service and copayment, in which patients bear part of the expenses of reimbursement. Similarly, the nonreimbursement cost includes all expenses for any nonreimbursed items. The out-of-pocket cost was estimated as the sum of the medical costs paid by the patient irrespective of reimbursement. All costs were calculated in Korean won and converted to US dollars (US $1=1200 Korean won).

**Definitions of cost categories for the patient-centered economic burden outcome.**

- Total medical cost: all expenses associated with each patient at the hospital, encompassing both reimbursed and nonreimbursed expenditures for 1 year (sum of the reimbursement and nonreimbursement costs)
- Reimbursement cost: all expenses incurred by patients restricted to items eligible for reimbursement for 1 year
- Nonreimbursement cost: all expenses incurred by patients restricted to items eligible for nonreimbursement for 1 year
- Out-of-pocket cost: all expenses borne directly by the patient irrespective of any reimbursement for 1 year

### Statistical Analysis

Data analyses were conducted using ATLAS (version 2.10.1; Observational Health Data Science and Informatics), the R software (version 4.0.3; R Foundation for Statistical Computing), the Health Resources Econometric Analysis Tool (HERMES), which we developed for cost analysis with the OMOP CDM vocabulary in R environment, and Health Analytics Data-to-Evidence Suite, formerly known as the OHDSI Methods Library. The HERMES code and algorithm are available on GitHub [19]. We evaluated the baseline characteristics of the exudative and nonexudative AMD groups, including demographics and clinical characteristics, drugs, and conditions. To reduce selection bias in the quasi-experimental design, we performed propensity score matching using regularized logistic regression with a Laplace prior (least absolute shrinkage and selection operator; LASSO) [20,21]. We used demographic data, conditions, drugs, procedures, and Charlson Comorbidity Index scores as covariates in the propensity score matching (Figure 1). We considered covariates as unmatched variables if the standardized difference was >0.10 after propensity score matching. We included single concepts and concept groups within the parent categories for conditions and drugs in the propensity score matching. We excluded highly correlated covariates related to exudative AMD, including visual- and retinal-related disorders, anti-VEGFs, verteporfin, and ophthalmological drugs. We used an exponential conditional model (ECM) with a generalized linear model to estimate the average cost by adjusting the confounding variable and overcoming the positive skewness of costs [22]. To determine a suitable distribution and link function, we performed modified Park tests and the Box-Cox test. The goodness of fit of the ECM was evaluated using the Akaike information criterion. Patients with 0 costs who did not incur costs during 1 year after the index date were excluded. We considered demographic data; 1-year costs before the index date, which were defined as the preindex costs; and unmatched variables from the propensity score matching as covariates to adjust for remaining confounders. To interpret the results, we exponentiated the coefficient. The exponentiated coefficient represents the expected change in the response variable associated with a 1-unit increase in the predictor variable when the other predictor variables are at their mean values.

### Ethical Considerations

This study was approved by the institutional review board of SNUBH (X-2012-657-902). The data were deidentified and anonymized; thus, informed consent was not required.

## Results

### Patient Characteristics

**Figure 2:**
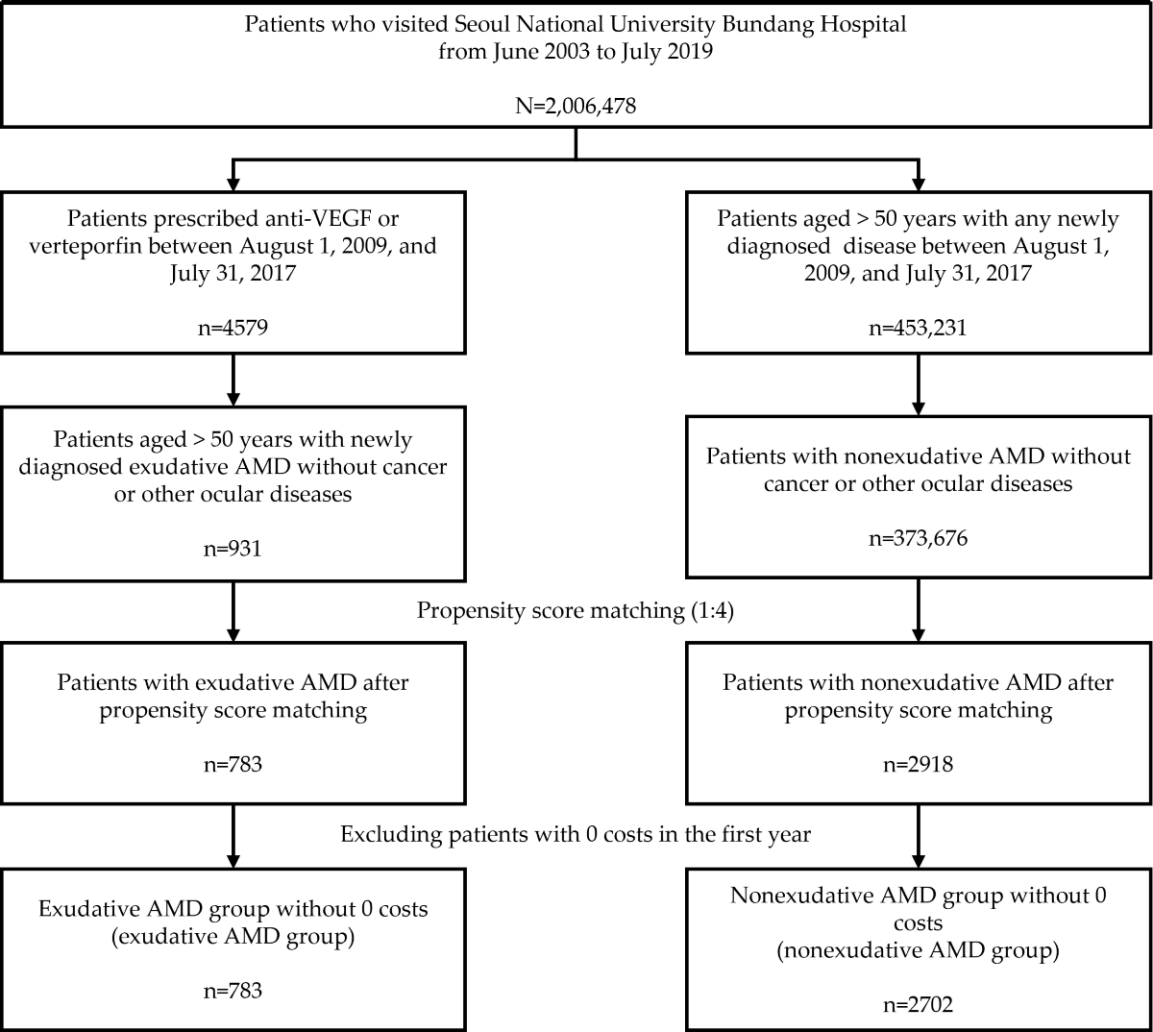
Selection flow of patients with exudative age-related macular degeneration (AMD) and nonexudative AMD from June 2003 to July 2019. VEGF: vascular endothelial growth factor.

Of the 2,006,478 patients who visited SNUBH, 931 (0.05%) were newly diagnosed with exudative AMD, and 373,676 (18.62%) had nonexudative AMD. After 1:4 propensity score matching, 84.1% (783/931) of patients with exudative AMD and 0.78% (2918/373,676) of patients with nonexudative AMD were matched. After excluding patients with 0 costs in the first year, of the 2918 patients with nonexudative AMD, there were 2702 (92.6%) left (Figure 2).

In the propensity score model, 1079 covariates were matched. The standardized mean difference was <0.10 for all variables that significantly affected medical costs, such as cardiovascular disease and opioids. In total, 4 covariates (“other antiepileptics”; “bisphosphonates, combinations”; “corticosteroids, potent, other combinations”; and “drug affecting bone structure and mineralization”) had a standardized mean difference of >0.10; thus, we included these variables in the ECM to adjust for remaining confounders (Table 1).

**Table 1:** Baseline demographic and clinical characteristics for the exudative age-related macular degeneration (AMD) group and nonexudative AMD group in the index period from August 2009 to July 2017 before and after propensity score (PS) matching.

Characteristic^a^			Before PS matching							After PS matching				
			Exudative AMD group (n=931)		Nonexudative AMD group (n=373,676)		SMD^b^		Exudative AMD group (n=783)			Nonexudative AMD group (n=2918)		SMD
Age (years), mean (SD)			72.47 (8.44)		62.48 (9.87)		1.01		71.52 (8.28)			72.16 (10.53)		−0.06
**Sex, n (%)**														
	Female	425 (45.65)		215,184 (57.59)		−0.24		348 (44.44)			1205 (40.55)		0.08	
**Medical history—general, n (%)^c^**														
	Acute respiratory disease	3 (0.32)		1144 (0.31)		0.00		1 (0.13)			7 (0.22)		−0.02	
	Chronic liver disease	6 (0.64)		3499 (0.94)		−0.03		3 (0.38)			6 (0.42)		0.00	
	Chronic obstructive lung disease	8 (0.86)		306 (0.22)		0.09		4 (0.51)			7 (0.22)		0.05	
	Dementia	16 (1.72)		4572 (1.22)		0.04		8 (1.02)			21 (0.67)		0.04	
	Depressive disorder	8 (0.85)		3542 (0.95)		−0.01		4 (0.51)			9 (0.29)		0.04	
	Diabetes mellitus	63 (6.77)		11,633 (3.11)		0.17		31 (4)			105 (4)		0.00	
	Gastroesophageal reflux disease	11 (1.18)		5942 (1.59)		−0.04		6 (0.77)			24 (0.82)		−0.01	
	Gastrointestinal hemorrhage	3 (0.32)		1751 (0.47)		−0.02		1 (0.13)			3 (0.11)		0.01	
	Hyperlipidemia	32 (3.44)		8838 (2.37)		0.06		19 (2.43)			63 (2.54)		−0.01	
	Hypertensive disorder	63 (6.77)		18,839 (5.04)		0.07		29 (3.7)			90 (3.61)		0.00	
	Lesion of liver	4 (0.43)		3123 (0.84)		−0.05		1 (0.13)			6 (0.33)		−0.04	
	Obesity	2 (0.21)		1155 (0.31)		−0.02		2 (0.26)			6 (0.19)		0.01	
	Osteoarthritis	16 (1.72)		13,706 (3.67)		−0.12		6 (0.77)			30 (1.4)		−0.06	
	Pneumonia	16 (1.72)		2883 (0.77)		0.09		10 (1.28)			23 (0.77)		0.05	
	Renal impairment	11 (1.18)		1904 (0.51)		0.07		6 (0.77)			13 (0.56)		0.03	
	Rheumatoid arthritis	3 (0.32)		1959 (0.52)		−0.03		2 (0.26)			11 (0.36)		−0.02	
	Viral hepatitis C	3 (0.32)		596 (0.16)		0.03		1 (0.13)			1 (0.03)		0.03	
**Medical history—cardiovascular disease, n (%)^c^**														
	Atrial fibrillation	13 (1.4)		2702 (0.72)		0.07		6 (0.77)			26 (0.84)		−0.01	
	Cerebrovascular disease	41 (4.4)		21,165 (5.66)		−0.06		26 (3.32)			67 (2.72)		0.04	
	Coronary arteriosclerosis	11 (1.18)		3866 (1.03)		0.01		3 (0.38)			14 (0.55)		−0.03	
	Heart disease	52 (5.59)		16,624 (4.45)		0.05		22 (2.81)			82 (3.22)		−0.02	
	Heart failure	5 (0.54)		1501 (0.4)		0.02		2 (0.26)			6 (0.29)		−0.01	
	Ischemic heart disease	17 (1.83)		6067 (1.62)		0.02		10 (1.28)			36 (1.54)		−0.02	
	Peripheral vascular disease	1 (0.11)		193 (0.05)		0.02		1 (0.13)			1 (0.03)		0.03	
**Medication use, n (%)^c^**														
	Agents acting on the renin-angiotensin system	83 (8.92)		18,055 (4.83)		0.16		39 (4.98)			138 (5.23)		−0.01	
	Antibacterials for systemic use	11 (1.18)		7935 (2.12)		−0.07		10 (1.28)			29 (1.09)		0.02	
	Antidepressants	44 (4.73)		15,864 (4.25)		0.02		24 (3.07)			79 (2.89)		0.01	
	Antiepileptics	40 (4.3)		11,917 (3.19)		0.06		19 (2.43)			72 (2.61)		−0.01	
	Anti-inflammatory and antirheumatic products	134 (14.39)		47,361 (12.67)		0.05		61 (7.79)			243 (10.39)		−0.09	
	Antithrombotic agents	124 (13.32)		31,233 (8.36)		0.16		62 (7.92)			238 (9.16)		−0.04	
	Beta-blocking agents	51 (5.48)		14,149 (3.79)		0.08		25 (3.19)			82 (3.16)		0.00	
	Calcium channel blockers	92 (9.88)		20,166 (5.4)		0.17		46 (5.87)			170 (6.69)		−0.03	
	Diuretics	67 (7.2)		13,486 (3.61)		0.16		31 (3.96)			111 (4.04)		0.00	
	Drugs for acid-related disorders	113 (12.14)		60,875 (16.29)		−0.12		60 (7.66)			207 (8.15)		−0.02	
	Drugs for obstructive airway diseases	29 (3.11)		7911 (2.12)		0.06		17 (2.17)			50 (2)		0.01	
	Drugs used in diabetes	54 (5.8)		11,107 (2.97)		0.14		26 (3.32)			110 (4.38)		−0.06	
	Immunosuppressants	3 (0.32)		1161 (0.31)		0.00		1 (0.13)			6 (0.29)		−0.04	
	Lipid-modifying agents	93 (9.99)		23,533 (6.3)		0.14		49 (6.26)			164 (6.15)		0.00	
	Opioids	67 (7.2)		36,821 (9.85)		−0.10		36 (4.6)			138 (5.23)		−0.03	
	Psycholeptics	102 (10.96)		45,363 (12.14)		−0.04		57 (7.28)			192 (7.95)		−0.03	
	Psychostimulants, agents used for ADHD^d^, and nootropics	8 (0.86)		2093 (0.56)		0.04		4 (0.51)			14 (0.54)		0.00	
	Other antiepileptics^e,f^	51 (5.48)		9697 (2.6)		0.15		19 (2.43)			116 (4.34)		−0.11	
	Drugs affecting bone structure and mineralization^f^	87 (9.34)		15,650 (4.19)		0.21		46 (5.87)			181 (8.63)		−0.11	
	Bisphosphonates (combinations)^f^	87 (9.34)		15,640 (4.19)		0.21		46 (5.87)			181 (8.63)		−0.11	
	Corticosteroids (potent; other combinations)^f^	64 (6.87)		11,736 (3.14)		0.178		33 (4.21)			127 (6.83)		−0.11	

^a^These covariates are a subset of the 1079 covariates used for PS matching.

^b^SMD: standardized mean difference.

^c^Medical history and medication use were identified through diagnosis and prescription within 1 year before the index date.

^d^ADHD: attention-deficit/hyperactivity disorder.

^e^Medication use was identified through prescription any time before the index date.

^f^The covariates were considered unmatched covariates based on standardized differences, which were adjusted in the following exponential conditional model.

### The Incremental Health Care Cost of Exudative AMD After Propensity Score Matching

The total medical costs observed in the first year were US $4565 in the exudative AMD group, US $3253 higher than those in the nonexudative AMD group. As a result of the ECM adjusting for remaining confounders, the total medical costs in the exudative AMD group were estimated to be US $4738, which was US $3426 more than those of the nonexudative AMD group. The reimbursement costs in the exudative AMD group were US $4038, which was approximately US $3130 more than the reimbursement costs incurred in the nonexudative AMD group. Regarding the nonreimbursement costs, those incurred in the exudative AMD group were US $776, which was US $366 more than the nonreimbursement costs in the nonexudative AMD group. The out-of-pocket costs were US $1241 in the exudative AMD group, which was US $680 higher than those in the other group. In the second year, the exudative AMD group also had higher medical costs than the nonexudative AMD group. In the ECM estimation, the total medical costs were US $1829 higher in the exudative AMD group. The exudative AMD group had higher reimbursement, nonreimbursement, and out-of-pocket costs of US $1461, US $373, and US $507, respectively (Table 2).

**Table 2:** The observed and estimated incremental health care costs of the exudative age-related macular degeneration (AMD) group compared with the nonexudative AMD group for 2 years after the index date.

		Observed costs after propensity score matching (US $)				Estimated costs from exponential conditional model^a^ (US $)		
		Exudative AMD group (n=783^b^), mean (SD)	Nonexudative AMD group (n=2702^b^), mean (SD)	Incremental costs	Exudative AMD group (n=783^b^), mean (SE)		Nonexudative AMD group (n=2702^b^), mean (SE)	Incremental costs
**First year**								
	Total costs	4564.86 (2702.37)	1311.86 (3201.04)	3253	4738.49 (127.42)		1312.22 (62.14)	3426.27
	Reimbursement costs	3784.40 (2313.04)	903.28 (2591.63)	2881.12	4038.44 (195.37)		908.42 (54.08)	3130.02
	Nonreimbursement costs	780.46 (731.74)	408.58 (853.27)	371.88	776.46 (23.31)		410.52 (15.73)	365.94
	Out-of-pocket costs	1244.21 (1470.13)	677.80 (1233.55)	566.41	1240.54 (46.56)		680 (22.77)	560.53
**Second year**								
	Total costs	1814.35 (2804.95)	418.57 (2548.41)	1395.79	2489.11 (1845.74)		659.91 (538.96)	1829.20
	Reimbursement costs	1385.39 (2336.94)	311.03 (2240.33)	1074.36	2036.23 (747.24)		575.50 (520.54)	1460.73
	Nonreimbursement costs	428.96 (107.54)	107.54 (525.1)	321.42	491.74 (92.01)		119.08 (21.65)	372.66
	Out-of-pocket costs	624.45 (1070.29)	194.36 (760.08)	430.09	736.81 (109.57)		229.91 (46.04)	506.90

^a^The exponential conditional model used a log link function with gamma distribution adjusting for sex, age, preindex cost, and unmatched covariates (“other antiepileptics”; “drugs affecting bone structure and mineralization”; and “corticosteroids, potent, other combination”). The SEs of the estimated costs were calculated using bootstrapping.

^b^After propensity score matching, patients with 0 costs in the first year were excluded.

### The ECM to Estimate the Patient-Centered Economic Burden of Exudative AMD After Propensity Score Matching

In the ECM, the total medical costs, reimbursement costs, nonreimbursement costs, and out-of-pocket costs in the first year were significantly 3.52, 4.25, 2.05, and 1.89 times higher, respectively, in the exudative AMD group. There were more significant differences in all costs in the second year than in the first year. The total medical costs, reimbursement costs, nonreimbursement costs, and out-of-pocket costs were significantly 4.93, 5.09, 4.68, and 3.50 times higher, respectively, in the exudative AMD group. Age was found to be a significant predictor of health care costs in the first year (*P*<.001) but not in the second year (*P*=.14). Sex was not a significant factor for costs in the ECM. The preindex cost was always a positive estimate and showed significance except for the nonreimbursement costs and out-of-pocket costs in the first year. Most of the unmatched covariates did not show significance; however, there were cases of positive values with significance for antiepileptics and bone structure–affecting drugs and negative values with significance for corticosteroids (Table 3).

**Table 3:** The multivariable analysis of medical costs in the first and second years after the index date using the exponential conditional models with propensity score matching in patients with exudative age-related macular degeneration (AMD).

			Intercept^a^				Exudative AMD group^b^				Age^c^					Sex^d^					Preindex cost^e^					Drug group												
																										Other antiepileptics^f^					Drugs affecting bone structure and mineralization^g^					Corticosteroids (potent; other combinations)^g^		
			β		*P* value		β		*P* value		β		*P* value		β			*P* value		β			*P* value		β			*P* value		β			*P* value		β			*P* value
**First year**																																						
	Total costs	6.0145		<.001		1.2646		<.001		.0147		<.001		.0984			.16		.0001			.002		.1163			.54		.2520			.16		−.0563			.59	
	Reimbursement costs	5.4161		<.001		1.4464		<.001		.0172		<.001		.1395			.09		.0001			<.001		.0884			.68		.2408			.24		.0175			.88	
	Nonreimbursement costs	5.3879		<.001		.7172		<.001		.0084		.007		.0165			.79		<.0001			.63		.1528			.36		.4959			.002		−.3160			<.001	
	Out-of-pocket costs	5.7831		<.001		.6345		<.001		.0095		.004		.0621			.28		<.0001			.09		.2131			.16		.3154			.03		−.1780			.04	
**Second year**																																						
	Total costs	4.8186		<.001		1.5948		<.001		.0156		.14		−.1408			.45		.0002			.004		1.0024			.04		−.1456			.76		−.2252			.41	
	Reimbursement costs	4.3207		<.001		1.6278		<.001		.0182		.10		−.2146			.34		.0002			.01		1.0219			.09		−.2270			.69		−.1129			.73	
	Nonreimbursement costs	4.5257		<.001		1.5430		<.001		.0008		.92		.0424			.78		.0001			.01		.9547			.02		.17			.66		−.5841			.01	
	Out-of-pocket Costs	4.9482		<.001		1.2527		<.001		.0032		.66		−.0294			.82		.0002			<.001		.8716			.10		.1453			.65		−.3055			.10	

^a^The exponential conditional model used a log link function with gamma distribution adjusting for sex, age, preindex cost, and unmatched covariates.

^b^The reference group was the nonexudative AMD group.

^c^Age was reported in years and is presented as a continuous variable in this table.

^d^Sex was represented as a binary variable, with a value of 1 indicating male and a value of 0 indicating female.

^e^Preindex cost was calculated for 1 year before the index date.

^f^Any time before index date.

^g^For −365 days to index date.

## Discussion

### Principal Findings

In this study, we found that patients with exudative AMD had a significantly higher economic burden than patients with nonexudative AMD over a 2-year follow-up period, particularly in terms of nonreimbursement and out-of-pocket costs. To address potential bias and confounding from real-world data, we used propensity score matching with LASSO and ECM. The findings of this study are notable as practically nothing is known about the long-term economic burden of exudative AMD from the perspective of patients (eg, nonreimbursement and out-of-pocket costs) using real-world data. The economic burden of patients with exudative AMD was significantly higher than that of patients with nonexudative AMD for 2 years. Specifically, the total medical costs adjusted for confounders after propensity score matching of the exudative AMD group were US $3426 and US $1829 in the first and second years, respectively. The incremental costs of reimbursement for exudative AMD were US $3130 in the first year and US $1461 in the second year. The nonreimbursement cost was US $366 higher in the exudative AMD group in the first year, and the incremental cost increased to US $373 in the second year. In addition, the out-of-pocket cost of exudative AMD was US $561 higher in the first year and continued to US $507 in the second year. To the best of our knowledge, this is the first study to derive real-world evidence of the patient-centered economic burden of exudative AMD. Our findings highlight the need for improved reimbursement strategies that reduce out-of-pocket costs for patients with exudative AMD, thereby reducing the financial burden associated with this disease.

### Comparison With Previous Studies

Historically, the economic burden of exudative AMD has been reported using various methods and outcomes but has not adequately captured the patient perspective using real-world data (Table 4). The annual disease burden of AMD was reported to be €51.3 to €101.1 million (US $54.8-$108.01 million) in France, Germany, Italy, and the United Kingdom in the study by Bonastre et al [9] and €5300 to €12,445 (US $5662.47-$13,296.10) in Canada, Germany, France, Spain, and the United Kingdom in the study by Cruess et al [11]. The annual cost of exudative AMD was 7 times higher than that of the control group in the study by Lotery et al [14]. In addition, high societal costs associated with exudative AMD were reported in the United States in the study by Brown et al [10]. These 4 studies were conducted using a survey, in which the burden of exudative AMD was very high even before the anti-VEGF era. After anti-VEGF therapy appeared, 3 studies reported the burden of the disease. Spooner et al [15] reported that the direct medical cost of exudative AMD was Aus $199.20 (US $126.66) and the indirect cost was Aus $64.8 (US $41.20) using questionnaires. Previous studies using self-reported measures or surveys can provide valuable information, but they have several limitations [23]. Research based on human interaction is subject to recall bias. Respondents may not remember or respond accurately, and it can be difficult to derive representative values. These are limitations that can lead to inaccurate results. Depending on the nature of the survey, there may be a limited amount of time to respond, and some questions may be difficult to answer, resulting in missing or inaccurate information. Furthermore, as it is impossible to survey the entire population, a survey may not be representative and may be subject to selection and social desirability biases, where respondents may underreport certain behaviors or overreport others because of perceived social norms. Finally, survey results may not be generalizable to other populations or periods.

**Table 4:** Previous studies associated with the economic burden of exudative age-related macular degeneration.

Study, year	Economic analysis		Outcomes
	Price	Quantity	
Bonastre et al [9], 2002	Unit cost	Consultation with an ophthalmologist	Yearly budget impact on medical cost
Lotery et al [14], 2007	Unit cost	Survey	Annual health resource use cost
Cruess et al [11], 2008	Unit cost	Self-reported and medical chart	Annual societal cost
Brown et al [10], 2016^a^	Self-reported weighted average annual cost from cohort	Self-reported weighted average annual cost from cohort	Annual direct and indirect costs
Spooner et al [15], 2018	Interview	Interview	Annual direct and indirect costs
Kim et al [13], 2019^a^	Exponential conditional model with generalized linear model using South Korean claims data	Exponential conditional model with generalized linear model using South Korean claims data	Incremental reimbursement cost
Jeon et al [12], 2020^a^	Generalized linear model using South Korean claims data	Generalized linear model using South Korean claims data	1-year reimbursement cost
Almony et al [8], 2021^a^	Generalized linear model using US claims data	Generalized linear model using US claims data	1-year reimbursement cost

^a^Economic burden was estimated using a macro-costing approach.

In contrast, analyzing real-world data can provide a more accurate and comprehensive economic burden reflecting clinical practice. The claims, EMR, and other administrative data can be more representative of the entire population and less prone to biases. There were several previous studies analyzing the burden of exudative AMD using real-world data. In the study by Kim et al [13] analyzing administrative data from South Korea, the total reimbursement cost of exudative AMD was 2.13 times to 4.06 times higher than that of nonexudative AMD, and the incremental reimbursement cost was estimated to be US $3699 through the ECM in South Korea. They compared controls as a nonhealthy population with comparable comorbidities using a propensity score model. The methodology overcame the limitations of cost data through ECM and generalizability by using the general population, and a significant 1-year economic burden was identified despite applying a conservative approach through the propensity score matching technique. However, the limitations of using claims data are that they do not reflect uninsured treatment and, therefore, do not define patients with exudative AMD in the real clinical environment, do not reflect nonpayment items such as bevacizumab and optical coherence tomography in the economic burden, and do not estimate the economic burden of real patients. Jeon et al [12] compared exudative AMD with diabetic macular edema and reported only the reimbursement cost with a 1-year follow-up using claims data. Owing to the matching group and changes in reimbursement criteria with exudative AMD, the cost was estimated to be higher than that in the study by Kim et al [13]. The most recent study investigated annual reimbursement costs [8]. Almony et al [8] reported the economic burden of exudative AMD by choroidal neovascularization, and it is the only study to stratify the economic burden by severity, patient-eye level in commercially insured patients in the United States; however, it was a short-term study with 18 months of follow-up and did not compare exudative AMD with any other conditions. The lack of a control group makes comparisons difficult, and the presence of distorting outliers may have overestimated the economic burden. In addition, as in previous studies, the patient-centered economic burden, which comprises nonreimbursed items or out-of-pocket costs, was not calculated. As in previous studies, we also found a high economic burden of exudative AMD and verified its association with exudative AMD using real-world data by adjusting for selection bias and skewed data. Compared with previous studies that only included insurance-covered items and underestimated the patients’ economic burden, we included nonreimbursement and out-of-pocket costs that directly affect patients’ economic burden. Furthermore, as exudative AMD requires long-term management, we stratified the economic burden by the first and second year and found that there was still a considerable economic burden in the second year.

### Policies for Exudative AMD

The economic burden of patients with exudative AMD is substantial worldwide. To address this issue, governments worldwide have expanded the insurance coverage for exudative AMD. In the United States, Medicare covers 80% of the cost of macular degeneration screening and anti-VEGF injections such as aflibercept, ranibizumab, bevacizumab, and verteporfin for Medicare beneficiaries. Similarly, in the United Kingdom, evidence-based medical recommendations are provided through organizations such as the National Institute for Health and Care Excellence, and cost-effectiveness analysis is used to cover anti-VEGFs such as aflibercept and brolucizumab. However, bevacizumab, which is as effective as ranibizumab [4], relatively inexpensive, and cost-effective [24], has not been approved and reimbursed in the United Kingdom or South Korea. In the United States, covering bevacizumab led to US $17.3-billion savings for Medicare and patients from 2008 to 2015 [25].

In South Korea, the pricing system operates on a fee-for-service structure, with the national health insurance acting as a third-party payer covering a portion of medical fees for items eligible for reimbursement [26]. Inpatient care typically involves an out-of-pocket rate of up to 20%. Outpatient services exhibit variations in out-of-pocket rates based on the hospital type (clinics at 30%, hospitals at 40%, general hospitals at 50%, and tertiary hospitals at up to 60% of the reimbursement costs [27]). To mitigate the financial burden on patients with exudative AMD in South Korea, the government has taken proactive measures. Sustained reimbursement has been established for optical coherence tomography measurements and anti-VEGF treatments. In addition, an extended benefit coverage policy has been introduced, reducing cost sharing to 10% for patients with rare and incurable conditions such as exudative AMD. However, as this study showed, the out-of-pocket cost for patients with exudative AMD remains significantly high, and this remains true even in the second year. Therefore, there is a need for a sustained, efficient, and sufficient reimbursement strategy for a high economic disease burden that efficiently uses limited national health insurance finances while reducing the burden on patients. Strategies include discovering new drugs via randomized clinical trials or using real-world data, and approving as well as reimbursing affordable and effective drugs, like bevacizumab, based on cost-effectiveness studies. Such a strategy could help reduce the out-of-pocket cost for patients with exudative AMD; improve their access to treatment; and, ultimately, establish the financial health of insurance by lowering the reimbursement cost. To establish this strategy, real-world data and evidence are needed for diseases with economic burden and rare and intractable diseases, including cost and clinical data.

### Economic Burden Analysis Using the OMOP CDM

It is difficult to analyze claims or EMR data because of the sensitive patient information. However, the advent of the OMOP CDM has introduced an environment in which data can be analyzed without disclosing sensitive information. Therefore, many recent studies have analyzed and published EMR or claims data through the OMOP CDM. Previous studies [28-30] using the OMOP CDM have investigated mainly clinical outcomes. In this study, we conducted the first-ever analysis of economic data that had been transformed and validated into the OMOP CDM, and we found that the OMOP CDM could be used for economic analysis with HERMES. The OMOP CDM allowed us to verify the validity of the clinical and cost data and HERMES made it easy to conduct econometric analysis under the OMOP-CDM structure. Compared with previous studies, we could estimate the patient-centered economic burden in the long term by analyzing valid data on costs, including nonreimbursed items, such as bevacizumab, or measurements. By identifying patients who use nonreimbursable treatments and estimating the long-term patient-centered economic burden, our study has significant implications for policy and economic evaluation studies of expensive drugs for rare and incurable diseases such as exudative AMD. Overall, our research highlights the power of the OMOP CDM for unlocking new insights into health care economics and improving the lives of patients with serious medical conditions.

### Limitations

This study has a number of limitations. First, the data source for this study was the medical records from a single tertiary hospital, which may not be structured and representative of the entire population of patients with exudative AMD. Nonetheless, to address this issue, the data were transformed into the OMOP CDM and validated by experts in the fields of medicine, data science, and economics. Furthermore, as patients with exudative AMD are more likely to seek treatment in tertiary hospitals, the exudative AMD group is less likely to be overestimated. In addition, to account for selection bias and confounding variables, a propensity score model using LASSO and the ECM were applied, and all the results were found to be robustly significant. Second, we systematically excluded specific medical conditions, including cancer and other ophthalmic diseases treated with anti-VEGF agents, from the cohorts under investigation. Our research focused on the meticulous assessment of the patient-centered economic implications related to exudative AMD. Given the pronounced economic impact associated with cancer, the substantial medical expenditures attributable to cancer could potentially exert an undue influence on the overall economic burden analysis. This influence has the potential to introduce statistical anomalies and distortions within the econometric model, thereby warranting their exclusion from our study cohorts as potential outliers. Moreover, our intention was to delineate the population of real-world patients with exudative AMD with utmost precision. The exclusion of other ophthalmic conditions served the purpose of averting potential misspecifications in our analytical framework. Third, other potential confounding variables such as the type and status of patients’ insurance, lifestyle habits, and the stage of exudative AMD were absent from the data set. Therefore, we were unable to include unobserved confounding variables. As we adjusted for selection bias using propensity score matching with LASSO and assessed covariate balance through standardized mean differences, we presume that this limitation’s influence on the study findings was negligible. In addition, most of the population in South Korea is covered by national health care insurance services, with just 3% of patients supported by medical aid programs.

### Conclusions

In conclusion, exudative AMD had a significantly greater economic impact for 2 years on reimbursement, nonreimbursement, and out-of-pocket costs than nonexudative AMD after adjusting for baseline demographic and clinical characteristics. Although economic policies could relieve the economic burden of patients with exudative AMD over time, the out-of-pocket cost of exudative AMD was still higher than that of nonexudative AMD for 2 years. Our findings support the need for expanding reimbursement strategies for patients with exudative AMD given the significant economic burden faced by patients with incurable and fatal diseases both in South Korea and worldwide.

## Abbreviations

AMD: age-related macular degeneration
CDM: Common Data Model
ECM: exponential conditional model
EMR: electronic medical record
HERMES: Health Resources Econometric Analysis Tool
LASSO: least absolute shrinkage and selection operator
OHDSI: Observational Health Data Sciences and Informatics
OMOP: Observational Medical Outcomes Partnership
SNUBH: Seoul National University Bundang Hospital
VEGF: vascular endothelial growth factor

## Data Availability

As the Observational Medical Outcomes Partnership Common Data Model is a distributed research network, access to the data is restricted to internal private networks, and the data are not publicly available.

## References

[1] Guymer RH, Campbell TG (2023). Age-related macular degeneration. Lancet.

[2] Han X, Chen Y, Gordon I, Safi S, Lingham G, Evans J, Keel S, He M (2023). A systematic review of clinical practice guidelines for age-related macular degeneration. Ophthalmic Epidemiol.

[3] Studnička J, Říhová B, Rencová E, Rozsíval P, Dubská Z, Chrapek O, Kolář P, Kandrnal V, Demlová R, Pitrová Š, Řehák J (2013). Cost and effectiveness of therapy for wet age-related macular degeneration in routine clinical practice. Ophthalmologica.

[4] Nguyen CL, Oh LJ, Wong E, Wei J, Chilov M (2018). Anti-vascular endothelial growth factor for neovascular age-related macular degeneration: a meta-analysis of randomized controlled trials. BMC Ophthalmol.

[5] Kaiser SM, Arepalli S, Ehlers JP (2021). Current and future anti-VEGF agents for neovascular age-related macular degeneration. J Exp Pharmacol.

[6] Rosenberg D, Deonarain DM, Gould J, Sothivannan A, Phillips MR, Sarohia GS, Sivaprasad S, Wykoff CC, Cheung CM, Sarraf D, Bakri SJ, Chaudhary V (2023). Efficacy, safety, and treatment burden of treat-and-extend versus alternative anti-VEGF regimens for nAMD: a systematic review and meta-analysis. Eye (Lond).

[7] Wecker T, Grundel B, Reichl S, Stech M, Lange C, Agostini H, Böhringer D, Stahl A (2019). Anti-VEGF injection frequency correlates with visual acuity outcomes in pro re nata neovascular AMD treatment. Sci Rep.

[8] Almony A, Keyloun KR, Shah-Manek B, Multani JK, McGuiness CB, Chen C, Campbell JH (2021). Clinical and economic burden of neovascular age-related macular degeneration by disease status: a US claims-based analysis. J Manag Care Spec Pharm.

[9] Bonastre J, Le Pen C, Anderson P, Ganz A, Berto P, Berdeaux G (2002). The epidemiology, economics and quality of life burden of age-related macular degeneration in France, Germany, Italy and the United Kingdom. Eur J Health Econ.

[10] Brown MM, Brown GC, Lieske HB, Tran I, Turpcu A, Colman S (2016). Societal costs associated with neovascular age-related macular degeneration in the united states. Retina.

[11] Cruess AF, Zlateva G, Xu X, Soubrane G, Pauleikhoff D, Lotery A, Mones J, Buggage R, Schaefer C, Knight T, Goss TF (2008). Economic burden of bilateral neovascular age-related macular degeneration: multi-country observational study. Pharmacoeconomics.

[12] Jeon H, Lee H, Yoon D, Lee Y, Kim JH, Jee D, Shin J (2020). Burden of diabetic macular oedema in patients receiving antivascular endothelial growth factor therapy in South Korea: a healthcare resource use and cost analysis. BMJ Open.

[13] Kim S, Park SJ, Byun SJ, Park KH, Suh HS (2019). Incremental economic burden associated with exudative age-related macular degeneration: a population-based study. BMC Health Serv Res.

[14] Lotery A, Xu X, Zlatava G, Loftus J (2007). Burden of illness, visual impairment and health resource utilisation of patients with neovascular age-related macular degeneration: results from the UK cohort of a five-country cross-sectional study. Br J Ophthalmol.

[15] Spooner KL, Mhlanga CT, Hong TH, Broadhead GK, Chang AA (2018). The burden of neovascular age-related macular degeneration: a patient's perspective. Clin Ophthalmol.

[16] Overhage JM, Ryan PB, Reich CG, Hartzema AG, Stang PE (2012). Validation of a common data model for active safety surveillance research. J Am Med Inform Assoc.

[17] Huser V, DeFalco FJ, Schuemie M, Ryan PB, Shang N, Velez M, Park RW, Boyce RD, Duke J, Khare R, Utidjian L, Bailey C (2016). Multisite evaluation of a data quality tool for patient-level clinical data sets. EGEMS (Wash DC).

[18] Huser V, Kahn MG, Brown JS, Gouripeddi R (2018). Methods for examining data quality in healthcare integrated data repositories. Pac Symp Biocomput.

[19] Choi K (2023). HERMES: a health resources econometric analysis tool. GitHub.

[20] Suchard MA, Simpson SE, Zorych I, Ryan P, Madigan D (2013). Massive parallelization of serial inference algorithms for a complex generalized linear model. ACM Trans Model Comput Simul.

[21] Tian Y, Schuemie MJ, Suchard MA (2018). Evaluating large-scale propensity score performance through real-world and synthetic data experiments. Int J Epidemiol.

[22] Manning WG, Mullahy J (2001). Estimating log models: to transform or not to transform?. J Health Econ.

[23] Bauhoff S, Michalos AC (2014). Self-report bias in estimating cross-sectional and treatment effects. Encyclopedia of Quality of Life and Well-Being Research.

[24] van Asten F, Michels CT, Hoyng CB, van der Wilt GJ, Klevering BJ, Rovers MM, Grutters JP (2018). The cost-effectiveness of bevacizumab, ranibizumab and aflibercept for the treatment of age-related macular degeneration-a cost-effectiveness analysis from a societal perspective. PLoS One.

[25] Rosenfeld PJ, Windsor MA, Feuer WJ, Sun SJ, Frick KD, Swanson EA, Huang D (2018). Estimating medicare and patient savings from the use of bevacizumab for the treatment of exudative age-related macular degeneration. Am J Ophthalmol.

[26] Jung HW, Kwon YD, Noh J (2022). How public and private health insurance coverage mitigates catastrophic health expenditures in Republic of Korea. BMC Health Serv Res.

[27] Park S (2021). Medical service utilization and out-of-pocket spending among near-poor National Health Insurance members in South Korea. BMC Health Serv Res.

[28] Li X, Burn E, Duarte-Salles T, Yin C, Reich C, Delmestri A, Verhamme K, Rijnbeek P, Suchard MA, Li K, Mosseveld M, John LH, Mayer M, Ramirez-Anguita J, Cohet C, Strauss V, Prieto-Alhambra D (2022). Comparative risk of thrombosis with thrombocytopenia syndrome or thromboembolic events associated with different covid-19 vaccines: international network cohort study from five European countries and the US. BMJ.

[29] Seo SI, Park CH, You SC, Kim JY, Lee KJ, Kim J, Kim Y, Yoo JJ, Seo W, Lee HS, Shin WG (2021). Association between proton pump inhibitor use and gastric cancer: a population-based cohort study using two different types of nationwide databases in Korea. Gut.

[30] You SC, Rho Y, Bikdeli B, Kim J, Siapos A, Weaver J, Londhe A, Cho J, Park J, Schuemie M, Suchard MA, Madigan D, Hripcsak G, Gupta A, Reich CG, Ryan PB, Park RW, Krumholz HM (2020). Association of ticagrelor vs clopidogrel with net adverse clinical events in patients with acute coronary syndrome undergoing percutaneous coronary intervention. JAMA.

